# Structural Elucidation and Anti-Tumor Activity of a Polysaccharide (CP2-S) from *Cordyceps militaris* Fruit Bodies

**DOI:** 10.3390/polym16141972

**Published:** 2024-07-10

**Authors:** Lina Zhu, Jinyan Wang, Qingjiu Tang, Yanfang Liu

**Affiliations:** 1 Institute of Edible Fungi, Shanghai Academy of Agricultural Sciences, Shanghai 201403, China; lenazhu@alumni.sjtu.edu.cn (L.Z.); wangjinyan@saas.sh.cn (J.W.); tangqingjiu@saas.sh.cn (Q.T.); 2 School of Agriculture and Biology, Shanghai Jiao Tong University, Shanghai 200240, China

**Keywords:** *Cordyceps militaris*, polysaccharide, structure, anti-tumor

## Abstract

A polysaccharide (CP2-S), consisting of glucose with a weight average molecular weight of 5.9 × 10^6^, was purified from the fruit bodies of *Cordyceps militaris*. In this work, the corresponding structure and anti-tumor activity in vivo were investigated. Methylation and NMR analysis revealed that CP2-S was composed of a →4)-α-D-Glc*p*-(1→ backbone with partial substitution occurring at *O*-6 by T-linked α-D-Glc*p* in every ten residues, which has not been reported in previous reports. In vivo anti-tumor experiments showed that CP2-S could inhibit the growth of Lewis lung carcinoma in mice. Tumor inhibition rates were 17.8%, 24.5%, and 29.5% at dosages of 12.5, 50, and 100 mg/kg/d, respectively. Compared with the cisplatin group, mice treated with CP2-S exhibited a significant increase in spleen index (increased 22.7–42.4%) and thymus index (increased 47.7–36.8%). Additionally, serum levels of IgM and IgG in tumor-bearing mice increased by approximately 6.11~10.75-folds and 1.31~1.38-folds, respectively. These findings prove that CP2-S significantly inhibited the growth of Lewis lung carcinoma through immune-enhancing activity in mice.

## 1. Introduction

*Cordyceps militaris* (*C. militaris*), a unique mushroom in East Asian countries, has been widely used as a traditional nourishing food in China for centuries [1,2]. Polysaccharides are the primary bioactive components in *C. militaris* with functions of immune regulation [3,4,5,6,7] and anti-tumor [8,9], antioxidant [6,10] and antivirus [11], anti-hyperlipidemia [12], hypoglycemic [13], anti-inflammatory [14], and anti-atherosclerotic [15] effects. To date, more than twenty kinds of polysaccharides have been isolated from *C. militaris* [16,17]. The majority of these polysaccharides consist of glucose, galactose, and mannose in different molar ratios. However, a few polysaccharides containing small ratios of rhamnose, arabinose, xylose, ribose, fucose, galacturonic acid, glucuronic acid, and N-acetyl galactosamine have also been isolated [16,17]. The chemical structures of polysaccharides are complex. Polysaccharides with different structures isolated from *C. militaris* might be related to the raw material, separation method, and purification method [1,2,3,5,6,7,8,9,10,15,17,18]. Over 220 reported structures of polysaccharides have been identified from *Ganoderma lucidum*, another traditional medicinal mushroom in Asian countries [19]. Compared with *G. lucidum*, the number of polysaccharides identified from *C. militaris* was relatively small. The activity of a polysaccharide is determined by its monosaccharide composition, molecular weight, glycosidic linkage, and degree of branching. Polysaccharides exhibit different biological activities due to their different structures. Discovering novel polysaccharides derived from *C. militaris* and investigating their bioactivities could significantly contribute to a deeper understanding of the relationships between polysaccharide structures and activities, as well as facilitate the development and utilization of *C. militaris*.

Natural polysaccharides derived from mushrooms not only have multiple physiological activities but also have the property of low toxicity or non-toxicity. Furthermore, their potential for chemical modification renders them invaluable in the research and development of natural medicines and health food products [20,21]. Though some biological activities of homogeneous polysaccharides from *C. militaris* have been reported, most of the experiments have been conducted in vitro [3,4,8,9,10,14,18,22,23,24,25,26], because it is difficult to obtain homogeneous polysaccharides in large quantities for animal experiments. In some reports, the biological activities of crude polysaccharides from *C. militaris* were assessed in vivo [8,12,27,28,29,30]. Whether the effect was caused by the polysaccharide was uncertain because the other components in crude polysaccharides might possess biological activities. Only a small portion of animal experiments were conducted using homogeneous polysaccharides from *C. militaris*, and these researches focused on immunomodulatory [5], anti-atherosclerotic [15], anti-hyperlipidemic [12,31,32,33], hypoglycemic [13,34], anti-allergic asthma [35], and hepatorenal protective effects [36]. For instance, Shang et al. successfully purified a polysaccharide from *C. militaris* named CBPS-II with a weight average molecular weight 1.27 × 10^3^ (a 1,3-branchedgalactomannoglucan featuring a linear backbone composed of (1→4)-linked α-d-glucopyranose) through Sevag precipitation and chromatography on a Sephadex G-100 column and investigated its hypoglycemic effect in diabetic mice [13]. Zhao et al. obtained a polysaccharide named AEPSa (Mw = 87.8 kDa, consisting of mannose, galactose, and glucose with the mole ratios of 2.2:15.1:1) from *C. militaris* and investigated its hypoglycemic activity and underlying mechanisms in high-fat diet and streptozotocin-induced T2DM mice [34]. Some previous studies have demonstrated the in vitro anti-tumor effects of *C. militaris* polysaccharides against various cell lines including A549, HT-29, HeLa, HepG2, K562, colon 205, PC-3 cells [9,26,37,38]. However, there is limited research on the in vivo anti-tumor activities of homogeneous polysaccharides obtained from *C. miliaris*.

The authors successfully isolated a novel polysaccharide (CP2-S) from *C. militaris* fruit bodies by hot water extraction, ethanol precipitation, DEAE-Sepharose Fast Flow, and Sephacryl S-400 high-resolution chromatography [39]. CP2-S had a weight average molecular weight of 5.9 × 10^6^ and was mainly composed of glucose. Immunostimulating experiments in vitro indicated that CP2-S could stimulate RAW264.7 macrophages to produce nitric oxide, secrete interleukin-1β and interleukin-2, and increase phagocytosis and respiratory burst activity, suggesting that CP2-S was a natural immunostimulating polysaccharide with potential for further application [39]. However, the structural characteristics of CP2-S have not been elucidated. It is well known that the molecular structure of polysaccharides is a very important issue in their biological activities. The bioavailability may be associated with structural patterns. Therefore, it is necessary to elucidate the structure of CP2-S and further investigate its biological activities. In this study, we elucidated the structural characteristics of CP2-S and assessed its anti-tumor activity in mice. The results may be helpful to improve our knowledge about the structural characteristics and anti-tumor activity of polysaccharides from *C. militaris*.

## 2. Materials and Methods

### 2.1. Materials and Regents

*C. militaris* fruit bodies were provided by Yunnan Institute of Botany (Kunming, China). Anti-IgG, anti-IgM, IgG, IgM, and 3,3′,5,5′-Tetramethybenzidine (TMB) were purchased from Sigma-Aldrich (St. Louis, MO, USA). Anti-mouse Ig was from BD Pharmigen (San Diego, CA, USA). Cisplatin was from Shandong Qilu Pharmaceutical Co., Ltd. (Jinan, China). All other reagents were from Chinese suppliers and of analytical grade.

### 2.2. Polysaccharide Preparation

**Figure 1:**
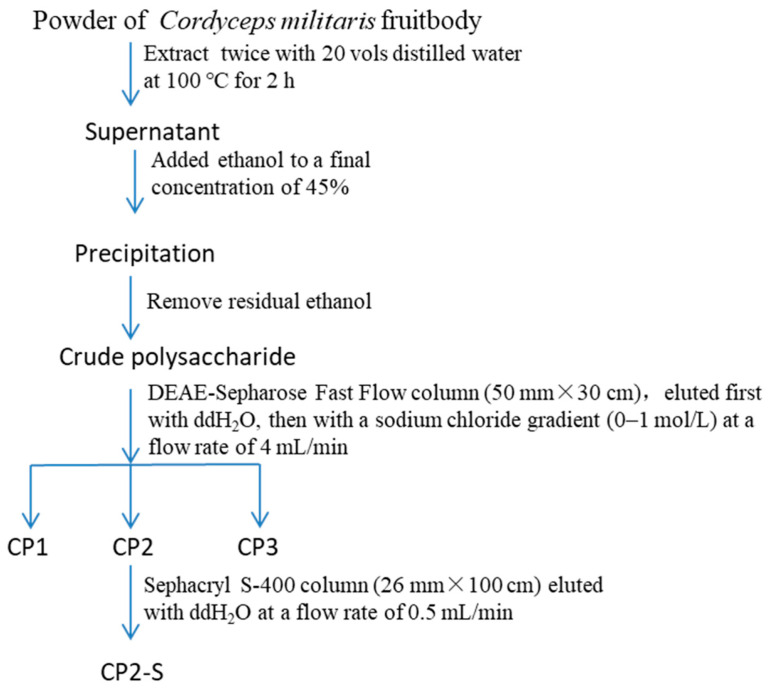
Preparation of polysaccharide (CP2-S) from *Cordyceps militaris* fruit bodies.

The polysaccharides (CP2-S) were extracted and isolated using the method described in our previous publication [39]. CP2-S from *C. militaris* fruit bodies were purified by hot water extraction, ethanol precipitation, DEAE-Sepharose Fast Flow, and Sephacryl S-400 high-resolution chromatography. The specific operating conditions for CP2-S purification are described in Figure 1.

### 2.3. Homogeneity, Molecular Weight, and Monosaccharide Composition Analysis

The homogeneity and average molecular weight (Mw) of polysaccharide CPS-2 were determined by high-performance size exclusion chromatography (HPSEC) on a Waters HPLC 2695 chromatographic system, which was equipped with a TSK-GEL G6000 PWXL column (7.8 × 300 mm), a 2414 refractive index detector (Waters, Milford, MA, USA), and a DAWN8^+^ light-scattering laser (Wyatt Corp., Santa Barbara, CA, USA). The monosaccharide composition of CP2-S was analyzed using high-performance anion-exchange chromatography with a pulsed amperometric detector (HPAEC-PAD) after hydrolysis with 3 mL of 2 mol/L trifluoroacetic acid.

### 2.4. Methylation and GC-MS Analysis

CP2-S was methylated following the established methods [26,27]. Dried CP2-S (2.5 mg) was dissolved in 0.5 mL of dimethyl sulfoxide by magnetic stirring at 85 °C for 2 h, then added with 20 mg NaOH and stirred for 3 h at room temperature, followed by adding 0.3 mL methyliodide and stirring in the dark at room temperature for 2.5 h. The mixture solution was added with deionized water to stop the reaction. The methylated polysaccharide was extracted using 3 mL of methylene chloride (CH_2_Cl_2_) and dried under a nitrogen stream. Methylated CP2-S was hydrolyzed with 0.5 mL of 4 mol/L trifluoroacetic acid (TFA) at 100 °C for 6 h. The hydrolsate was reduced with 3 mg sodium borodeuteride (NaBD_4_) for 12 h at room temperature, followed by acetylation with 0.5 mL acetic anhydride for 2 h at 100 °C. The partially methylated alditol acetate (PMAA) was analyzed by the GC–MS system (Thermo Finnigan TRACE 2000/MS, San Jose, CA, USA), equipped with a DB-5MS column (30 m × 0.25 mm × 0.25 μm) under a temperature program from 180 °C to 270 °C at 20 °C/min and kept at 270 °C for 25 min. The methylated polysaccharide linkages were identified by the retention time and fragmentation pattern.

### 2.5. NMR Analysis

CP2-S (30 mg) was dissolved in 1 mL of deuterium oxide for nuclear magnetic resonance (NMR) analysis using a 600 MHz Varian VNMRS NMR spectrometer (Agilent, Santa Clara, CA, USA). ^1^H NMR and ^13^C NMR spectra were recorded at 600 MHz and 150 MHz, respectively, at 70 °C. Homonuclear ^1^H-^1^H correlation spectroscopy (COSY), total correlation spectroscopy (TOCSY), heteronuclear single quantum correlation spectroscopy (HSQC), and heteronuclear multiple bond correlation spectroscopy (HMBC) were recorded using the standard Varian pulse sequence.

### 2.6. Effect of CP2-S on Lewis-Lung-Carcinoma-Bearing Mice In Vivo

#### 2.6.1. Animal and Experimental Design

A Lewis lung carcinoma cell line was provided by the State Key Lab of New Drug and Pharmaceutical Process at Shanghai Institute of Pharmaceutical Industry, China. Male C57BL/6 mice (weighing 18–20 g, SPF level) were purchased from Shanghai Laboratory Animal Center, CAS (SLACCAS) (Shanghai, China). Mice were randomly divided into 5 groups consisting of 10 mice each for different treatments. Each mouse was injected with 0.2 mL Lewis lung carcinoma cell suspension (2 × 10^7^ cells/mL) by subcutaneous injection into the hypodermis of the forelimb armpit to induce tumors. CP2-S solutions were administrated to mice at selected doses via intraperitoneal injection daily from day 1 to day 14 after tumor cell inoculation. Mice in the cisplatin group were injected with cisplatin at a dosage of 2 mg/kg/d, and mice in the control group were injected with physiological saline. The use of experimental animals follows the guidelines of the ethical committee of Shanghai Institute of Pharmaceutical Industry.

#### 2.6.2. Determination of Tumor Inhibition Rate, Spleen Index, and Thymus Index

Mice were weighed and sacrificed 24 h after the last drug administration. Spleens, thymuses, and tumors were removed and weighed. Spleen index, thymus index, and tumor inhibition rate were calculated. The anti-tumor efficacies of the treatments were assessed by calculating the percentage reduction in tumor weight compared with the control.

Spleen index = (spleen weight/mouse weight) × 10

Thymus index = (thymus weight/mouse weight) × 10

Tumor inhibition rate (%) = (tumor weight of the control group − tumor weight of the treatment group)/tumor weight of the control group × 100

#### 2.6.3. Determination of IgM and IgG Levels in Serum

Blood was sampled from each mouse and centrifugated at 10,000× *g* for 5 min to separate serum. IgM and IgG levels in serum were measured using enzyme-linked immunosorbent assay (ELISA) according to the method described by Zhang et al. [40].

## 3. Results

### 3.1. Structural Elucidation

#### 3.1.1. Homogeneity, Molecular Weight, and Monosaccharide Composition Analysis

HPSEC analysis results indicated that CP2-S was a homogeneous polysaccharide with a weight average molecular weight of 5.9 × 10^6^. An analysis of monosaccharide composition revealed that CP2-S was predominantly composed of glucose.

#### 3.1.2. FTIR and Methylation Analysis

**Figure 2:**
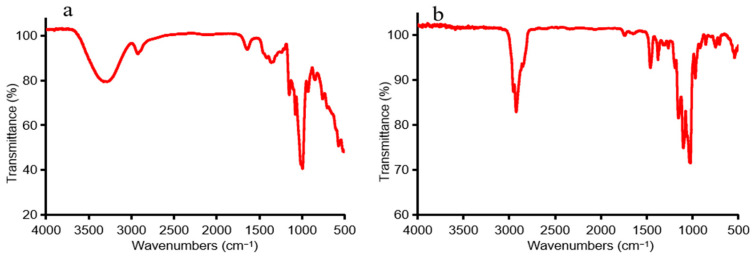
The FTIR spectra of the non-methylated and methylated CP2-S: (**a**) non-methylated, (**b**) methylated.

FTIR spectra of non-methylated and methylated CP2-S shown in Figure 2 were obtained using an FTIR spectrometer (Thermo Fisher Scientific, Waltham, MA, USA) with a range of 500–4000 cm^−1^. The complete methylation was confirmed by the disappearance of the –OH band (3100–3700 cm^−1^) in the IR spectrum. The individual peaks of the PMAA of CP2-S and the linkage patterns of fragments were identified through the analysis of mass spectra and the relative retention time in GC-MS. The percentage of methylated residues was estimated by calculating peak area ratios. Table 1 presents the results of methylation analysis, which revealed the presence of 1,5-di-O-acetyl-2,3,4,6-tetra-O-methyl-Glucitol, 1,4,5-tri-O-acetyl-2,3,6-tri-O-methyl-Gluitol, and 1,4,5,6-tetra-O-acetyl-2,3-di-O-methyl-Gluitol, respectively, in a molar ratio close to 1.00:10.14:0.97, indicating that CP2-S primarily consisted of terminal Glc*p*, 4-linked Glc*p*, and 4,6-linked Glc*p* residues.

#### 3.1.3. NMR Analysis

**Figure 3:**
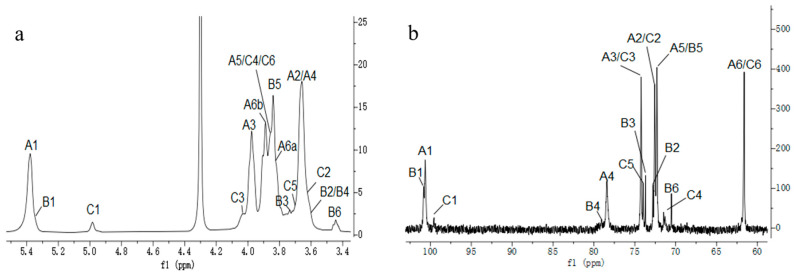
One-dimensional NMR spectra of CP2-S in D_2_O at 70 °C. (**a**) ^1^H NMR spectrum, (**b**) ^13^C NMR spectrum.

**Figure 4:**
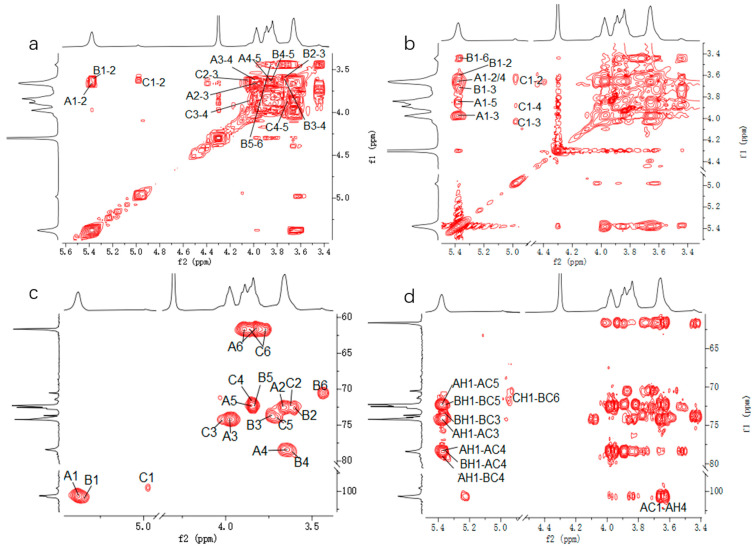
Two-dimensional NMR spectra of CP2-S in D_2_O at 70 °C. (**a**–**d**) represent COSY, TOCSY, HSQC, and HMBC spectra, respectively.

The structure of CP2-S was further elucidated through 1D and 2D NMR spectroscope. The ^1^H NMR spectrum (Figure 3a) of CP2-S revealed the presence of two anomeric proton signals at δ5.38 and 4.98 ppm, with a peak area ratio of approximately 10:1. The former were significantly overlapping but effectively distinguished at δ5.38 and 5.37 ppm through a comprehensive analysis of HSQC (Figure 4c) and TOCSY (Figure 4b). Signals were designated as residue A, B, and C, respectively. These signals also suggested the presence of α-configuration for glucopyranosyl residues [41]. Combined with the analysis of cross peaks in the HSQC spectrum (Figure 4c), the corresponding anomeric carbon signals of residues A, B, and C in ^13^C NMR (Figure 3b) were assigned at δ100.62, 100.83, and 99.57 ppm, respectively, indicating the presence of three α-configuration glucose residues existing in CP2-S [42,43]. The downfield shifts observed at δ 78.40 and 79.04 ppm indicated *O*-substitution at C-4 and were assigned to C-4 of the 4-linked α-D-Glc*p* and 4,6-linked α-D-Glc*p* based on methylation results and previous reports [44]. The signal observed at δ70.52 ppm corresponded to C-6 of 4,6-linked α-D-Glc*p*.

In terms of residue A, the chemical shift of H-2 was labelled at δ3.67 ppm based on the cross peak to resonance between H-1 and H-2 in the COSY spectrum (Figure 4a). The assignments for H-3, H-4, H-5, H-6a, and H-6b were determined by analyzing the cross signals in COSY and TOCSY spectra (Figure 4b). The identification of H-6a and H-6b was further supported by analysis of the HSQC spectrum (Figure 4c). Based on signal analysis in the HSQC spectrum, the chemical shifts of all carbon atoms in residue A were assigned accordingly. Comparison with the literature data [44] on the ^13^C and ^1^H chemical shifts, as well as methylation analysis, confirmed that residue A was a 4-linked α-D-Glc*p*.

For residue B and C, the chemical shift assignments of ^1^Hand ^13^C signals were determined in the same way as residue A. According to previous reports [43,44,45] and the results of methylation analysis, residue B was identified as a 4,6-linked α-D-Glc*p*, while residue C was designated as a terminal-linked α-D-Glc*p*. A summary of the ^1^H and ^13^C NMR spectral assignments for all residues can be found in Table 2.

HMBC was performed to analyze the backbone and the substitution sites within the repeating unit of polysaccharides. In Figure 4d, the strong cross peaks (5.38/78.40) indicated the correlation between H-1 of 4-linked α-D-Glc*p* and C-4 of the neighboring 4-linked α-D-Glc*p*. The cross peaks (3.64/100.62) represented the correlation between H-4 of 4-linked α-D-Glc*p* and C-1 of the neighboring 4-linked α-D-Glc*p*. The overlapped cross peaks (5.37/78.40) represented the correlation between H-1 of 4,6-linked α-D-Glc*p* and C-4 of the adjacent 4-linked α-D-Glc*p*. The cross peaks (5.38/79.04) represented the correlation between H-1 of 4-linked α-D-Glcp and C-4 of the neighboring 4,6-linked α-D-Glc*p*. An analysis of the aforementioned cross-peak signals revealed that CP2-S primarily consisted of the repeating units with a backbone composed of 4-linked α-D-Glc*p*. Finally, the cross peaks (4.98/70.52) indicated a correlation between H-1 of T-linked α-D-Glc*p* and C-6 of the neighboring 4,6-linked α-D-Glc*p*, suggesting that the side chain of CP2-S was composed of T-linked α-D-Glc*p* branching at *O*-6 of the backbone of 4-linked α-D-Glc*p*.

An analysis of the obtained data led us to conclude that CP2-S possessed a backbone composed of repeat units comprising 4-linked α-D-Glc*p*, with partial substitution occurring at *O*-6 by T-linked α-D-Glc*p* every ten residues of 4-linked α-D-Glc*p*. The deduced repeat unit of polysaccharide CP2-S was as follows:


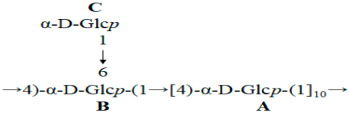


### 3.2. Effect of CP2-S on Lewis-Lung-Carcinoma-Bearing Mice In Vivo

**Figure 5:**
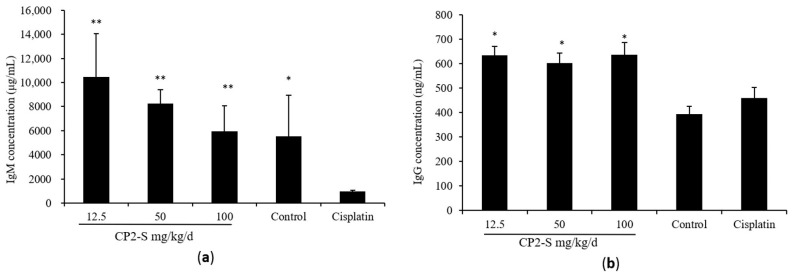
Effect of CP2-S on the concentration of IgM (**a**) and IgG (**b**) in mice serum: Blood from 5 mice in each group was sampled randomly. Note: * and ** indicate significant difference compared with cisplatin at *p* < 0.05 and *p* < 0.01, respectively.

The anti-tumor effect of CP2-S was investigated through intraperitoneal administration in Lewis-lung-carcinoma-bearing mice. Tumor inhibition rates recorded for CP2-S at dosages of 12.5, 50, and 100 mg/kg/d were 17.8%, 24.5%, and 29.5%, respectively (Table 3). Spleen and thymus are important immune organs. Spleen index and thymus index can reflect the immune function of the host. Compared with the cisplatin group, CP2-S significantly increased the spleen index (increased 22.7–42.4%) and thymus index (increased 47.7–36.8%) in Lewis-lung-carcinoma-bearing mice. Immunoglobulin is a crucial component of the immune system. Compared with the cisplatin group, serum levels of IgM and IgG in tumor-bearing mice increased by approximately 6.11~10.75-folds and 1.31~1.38-folds, respectively (Figure 5).

## 4. Discussion

Mushrooms contain bioactive polysaccharides, which exhibit varying chemical composition. The majority of these polysaccharides belong to the group of β-glucans, characterized by β-(1→3) linkages in their main chain and additional β-(1→6) branch points that are crucial for their anti-tumor activity [46]. Polysaccharides with high-molecular-weight glucans appear to be more effective than those with low molecular weight [46]. Some polysaccharides have been isolated from *C. militaris*, with reported molecular weights ranging from 4.3 to 47,960 kDa. Most of them are heteropolysaccharides with alpha or beta configurations [47]. The differences in these isolated polysaccharides might be contributed to strain origins, extraction methods, and purification procedures. The glycosidic bond is an important factor affecting the biological activities of polysaccharides. It was believed that β-glucan from *Ganoderma lucidum* possessed tumor activity, but recently, some reports have indicated that glucosyls in α-D-configuration also play a key role in the anti-tumor activity [48,49]. The polysaccharide CP2-S, derived from *C. militaris* fruit bodies, exhibited a backbone composed of (1→4)-linked-α- Glc*p* with partial substitution occurring at *O*-6 by T-linked α-D-Glc*p* every ten residues. Though polysaccharides with (1→4)-linked-α- Glc*p* backbone structure from *Cordyceps militaris*, *Cordyceps sinensis*, and *Cordyceps gunnii* have been reported, the structure of CP2-S was different in previous reports. Polysaccharides with (1→4)-linked-α- Glc*p* backbone exhibited immune-stimulatory and anti-tumor activity [7,50,51]. Most of the studies on structure characteristics and biological activities of *C. militaris* polysaccharides focused on low-molecular-weight polysaccharides (Mw under 50 kDa) [1,3,5,6,9,10,13,15,18,20,22,23,24,31,34,35]. He et al. reported a homogenous polysaccharide (average molecular weight 4.796 × 10^4^ kDa, consisting of glucose, manose, and galactose with the molar ratio of 8.09:1.00:0.25) from *C. militaris* fruit bodies significantly promoted macrophage phagocytosis and secretion of NO, TNF-α, and Il-6 in vitro [7]. CP2-S with a weight average molecular weight of 5.9 × 10^6^ was found to stimulate nitric oxide production, phagocytosis, respiratory burst activity, and the secretion of interleukin-1β and interleukin-2 of macrophages [39]. Our experiments indicated that glucosyls in α-D-configuration played a key role in the anti-tumor activity of *C. militaris* polysaccharides, and *C. militaris* polysaccharides with high molecular weight might possess remarkable bioactivities. Previous studies indicated *C. militaris* polysaccharides could inhibit various tumor cells in vitro [9,26,37,38]. The inhibition of other tumor cells needs further research, and polysaccharide modification of CP2-S can be conducted to enhance biological activities.

β-(1→3) or β- (1→6) glucan with anti-tumor and immunological activities existed in fungal cell walls [52]. Our previous study demonstrated that CP2-S could only be extracted from powdered and finely ground *C. millitaris* fruit bodies, which indicated that alpha-glucan existed in the cell walls. As biological response modifiers, fungal polysaccharides exerted their anti-tumor effects mainly by activating the immune response of host cells [53]. Unlike small-molecule anticancer drugs that directly kill cancer cells, polysaccharides derived from mushrooms usually have low toxicity and exert their anti-tumor effects through modulation or activation of the host immune responses [50]. Evidence suggests that mushroom polysaccharides possess immune-enhancing activity by stimulating natural killer cells, T-cells, B-cells, and macrophage-dependent immune system responses [3]. In vitro experiments demonstrated that CP2-S exhibited significant immunostimulatory effects by activating macrophages [39]. The present study indicated that CP2-S from *C. militaris* inhibited tumor growth in mice and stimulated the secretion of cytokines in vivo. Our data suggested that the anti-tumor activity of CP2-S was associated with its immune-enhancing properties, which was similar to those observed in other mushroom polysaccharides.

Furthermore, Bi et al. reported that CMPB90-1, a natural polysaccharide from *C. militaris,* exhibited the ability to modulate tumor-related macrophages (TAMs) by shifting their phenotype from a tumor-promoting M2 state to a tumor-killing M1 state [49]. In the tumor microenvironment, most of the macrophages had an M2-like phenotype, which was involved in immunological tolerance and tumor progression. An ideal method to target TAMs was not by depletion but rather by polarizing the M2 TAMs into the M1 phenotype, which then killed the tumor cells [54,55]. The current drugs targeting TAMs, including chemotherapeutic drugs, antibodies, or small-molecule inhibitors, were associated with potential adverse effects that affect all macrophage subsets, including the M1 macrophages and other immune cells [56]. Polysaccharides from *C. militairs* have the potential to be developed as safe drugs that specifically stimulate macrophages to participate in anti-tumor immune responses.

Sometimes achieving optimal results solely through the use of natural polysaccharides at low dosages can be challenging. In order to improve the biological activity and physiological function of these polysaccharides, studying on their synergistic effects has been one of the focuses of polysaccharide research in recent years. The main purpose of combination therapy is to enhance the efficacy of drugs by reducing adverse reactions or weakening drug resistance, thereby achieving better therapeutic effects. Due to the diverse biological activities and low toxicity of natural products, combination with clinical drugs has been increasingly used in disease treatment. Polysaccharides from *Letinula edodes* (lentinan) have been employed as an immunomodulator in the clinical treatment of tumors [57,58,59]. *C. militaris* polysaccharides exhibit multiple biological activities and minimal toxicity. The synergistic and detoxifying effects resulting from their combined usage with clinical drugs are also worthy of further research.

## 5. Conclusions

In this study, we reported the structure and anti-tumor activity of a homogeneous high-molecular-weight polysaccharide, designated as CP2-S, derived from *C. militaris* fruit bodies. CP2-S is composed of a →4)-α-D-Glc*p*-(1→ backbone with partial substitution occurring at O-6 by T-linked α-D-Glc*p* in every ten residues. In addition, in vivo experiments revealed that CP2-S effectively inhibited the growth of Lewis lung carcinoma in mice, enhanced the spleen index and thymus index of mice, and upgraded IgM and IgG levels in the serum of tumor-bearing mice. These findings suggested that CP2-S exerted its anti-tumor effect through enhancing the host immune response.

The findings are beneficial to illustrate the connection between the structure and biological activities of polysaccharides. Moreover, our study provided an effective scientific basis for the development of *C. militaris* polysaccharide as a functional diet and Chinese medicine product. Nevertheless, extensive studies should be carried out, such as investigating other potential activities, modifying polysaccharides to enhance their biological activity, exploring synergistic effects with drugs to improve efficacy, etc., which need to be addressed step by step.

## Figures and Tables

**Table 1:** GC-MS analysis of the PMAA of CP2-S.

PMAA	Type of Linkage	Molar Ratio	Major Mass Fragments (*m*/*z*)
1,5-Ac_2_-2,3,4,6-Me_4_-Glc*p*	Terminal-Glc*p*	1.00	87, 102, 118, 129, 145, 161, 162, 205
1,4,5-Ac_3_-2,3,6-Me_3_-Glc*p*	→4)-Glc*p*-(1→	10.14	87, 99, 102, 113, 118, 129, 131, 162, 173, 233
1,4,5,6-Ac_4_-2,3-Me_2_-Glc*p*	→4,6)-Glc*p*-(1→	0.97	85, 99, 102, 118, 127, 142, 159, 162, 187, 201, 261

**Table 2:** ^1^H and ^13^C NMR chemical shift data for CP2-S (δ, ppm).

Residues	Proton or Carbon					
	H-1/C-1	H-2/C-2	H-3/C-3	H-4/C-4	H-5/C-5	H-6/C-6
A: →4)-α-Glc*p*(1→	5.38	3.67	3.97	3.64	3.85	3.83 ^a^, 3.89 ^b^
	100.62	72.55	74.22	**78.40** *	72.29	61.61
B: →4,6)-α-Glc*p*(1→	5.37	3.60	3.73	3.61	3.84	3.44
	100.83	72.79	73.98	**79.04** *	72.29	**70.52** *
C: α-Glc*p*(1→	4.98	3.62	4.03	3.86	3.70	3.76 ^a^, 3.86 ^b^
	99.57	72.65	74.22	71.29	73.97	61.61

Note: * The bold values represent the chemical shift values of the substitution sites. ^a, b^ Interchangeable.

**Table 3:** Effect of CP2-S on tumor inhibition rate, spleen index, and thymus index on lung-carcinoma-bearing mice.

Group	Dosage (mg/kg/d)	Tumor Inhibition Rate (%)	Spleen Index	Thymus Index
CP2-S	12.5	17.8 ± 0.6	0.81 ± 0.04	0.26 ± 0.02
	50	24.5 ± 0.7	0.94 ± 0.06	0.26 ± 0.02
	100	29.5 ± 0.4	0.88 ± 0.05	0.28 ± 0.02
Cisplatin	2	78.3 ± 0.3	0.66 ± 0.03	0.19 ± 0.01
Control	physiological saline	0	1.00	1.00

Note: Data are the average values of the experiment. There are 10 mice in each group, and no mice died during the experiment.

## Data Availability

The data presented in this study are available on request from the corresponding author.
